# Management Control Systems and Clinical Experience of Managers in Public Hospitals

**DOI:** 10.3390/ijerph15040776

**Published:** 2018-04-17

**Authors:** Rogério Joao Lunkes, David Naranjo-Gil, Ernesto Lopez-Valeiras

**Affiliations:** 1 Department of Accounting, University Federal of Santa Catarina, Florianópolis-SC 88040-900, Brazil; 2 Financial Economics and Accounting Department, Pablo de Olavide University, 41013 Sevilla, Spain; dnargil@upo.es; 3 Financial Economics and Accounting Department, Universidad de Vigo, 32004 Ourense, Spain; elvaleiras@uvigo.es; 4 Biocost Research Group, Galicia Sur Health Research Institute (IIS Galicia Sur), SERGAS-UVIGO, 36312 Vigo, Spain

**Keywords:** clinical experience, managerial information, management control, public hospitals, PLS method

## Abstract

Healthcare authorities are encouraging managers in hospitals to acquire clinical experience and knowledge in order to better carry out and coordinate healthcare service delivery. The main objective of this paper is to analyse how the clinical experience of hospital managers is related to public health institutions’ performance. It is proposed that the effect of the clinical experience on operative and financial organizational performance is indirect through the mediating variables of perceived utility of management information and horizontal management control system. This paper analyses how these variables impact hospital performance through the data from a survey sent to 364 hospital managers in Brazil. The results show that managers’ clinical experience is related to higher perceived utility of historical, financial, short-term, and internal information, but not with horizontal control adoption in hospitals. Furthermore, our results show that, in hospitals, perceived utility of forecasted, non-financial, long-term, and external managerial information positively affects hospitals’ financial performance, while adoption of horizontal control management positively affects operational performance. Through showing evidence that clinical background could explain the differences not only in hospital service management but also in information capabilities and management control processes, this study offer meaningful implications for healthcare authorities and hospital managers involved in the development and implementation of strategies in the health sector.

## 1. Introduction

Under the new public management paradigm, hospital managers are required to deliver greater efficiency and more flexible public services. In several countries, for example, Brazil, formal legislation requires healthcare authorities to encourage hospitals to become more efficient organizations that respond to citizens’ demands with higher quality services at lower costs [1,2]. Hospitals are characterized as organizations with interdependent and complex activities, a lot of teamwork, and interconnectedness between health services. Thus, managers should implement managerial strategies that enhance control over clinical complexity by better allocating healthcare resources and coordination of services [3,4]. Several authors argue that the efficient allocation of resources requires a remarkable understanding of the capacities and resources of the organization, and such an understanding can only be developed over time through experience with human resources, organizational routines, and internalization of the common, tacit knowledge of the organization [5,6]. This implies that managers in hospitals should have clinical experience to encourage efficiency in carrying out activities, autonomy of work, and coordination in clinical service delivery. Along these lines, healthcare management researchers have argued that clinical experience of managers in hospitals can explain the differences not only in hospital service management but also in management control information capabilities and processes [6,7]. Such an approach, however, remains somewhat limited because of the multiple under-research dimensions of management control information.

To address efficiency and quality of service, managers need the information provided by management control systems. These systems can comprise all techniques and routines of a hospital to ensure efficient and proper managerial behaviour of organizational participants [3,8,9]. Management control systems may provide managers with different types of data related to performance, such as narrowly oriented information (i.e., historical, financial, short-term, and internal) and broadly oriented information (i.e., forecasted, non-financial, long-term, and external) [10]. Although it has been suggested that management control information may be useful for facilitating resource allocation and performance evaluation, there is limited empirical research that thoroughly examines its role in hospitals.

Research in health management posits that an important reason hospital managers do not deliver improved performance on many occasions is that vertical management control systems (e.g., rules and standard operating procedures, standard operating budget) work less effectively in organizations as complex as hospitals [11,12]. Vertical management controls are based on standard operating rules and procedures, and are focused on the measurement and evaluation of results. Thus, they foster competition instead of promoting cooperative behaviours among healthcare services, which often have interdependent activities. Conversely, horizontal management control is focused on fostering social control, which promotes autonomy and mutual collaboration, participation among different organizational levels, and greater interaction and efficiency in organizational relationships [5,12].

The general objective of this paper is to analyse how the clinical experience of hospital managers is related to public health institutions’ performance through perceived utility of managerial information and horizontal management control system. This paper reports results from a larger research project about public hospital management in Brazil. Brazil offers a mixed financed healthcare system (public and private). The universal public healthcare system includes approximately 30% of the total hospitals. It is financed, by law, with at least 15% of the net current national revenue.

## 2. Literature Background

Healthcare management research has shown that clinical experience is related to years of activities in medical care and healthcare management, especially in hospitals [7,13,14]. Clinical experience is a key variable affecting healthcare management, since experience is related to autonomy, and reliability is related to better performance in clinical activities and tasks [15]. Empirical evidence suggests that clinical experience is positively associated with increased engagement in quality healthcare improvement initiatives and strategic decisions [16]. It is also important to develop horizontal management controls [6].

The healthcare management literature provides evidence about the importance of the perceived utility of information for managing uncertainty and complexity in hospitals and others healthcare organizations. Recently, several authors have shown that managers believe in control systems that provide greater information detail to management healthcare organizations, since those systems provide more relevant, richer, and useful data [11,17]. Furthermore, Naranjo-Gil and Hartmann [18] suggest that perceived utility of wide scope systems affects the way information is used in healthcare organizations. Thus, hospitals with such systems are significantly more profitable, generating greater cash flows, and they have proportionately lower administrative expenses [19]. In this respect, Ologeanu-Taddei et al. [17] have shown that managers perceived a broad design of hospital information systems as very useful, since this type of information helps them to reduce medical risk in the prescription process for anesthesiologists, surgeons, and physicians.

Nowadays, hospitals are pushed to adopt horizontal-control management information systems, which focus on control between multiple units and tasks in lateral relations, which is highly relevant for hospitals as professional service organizations [11]. Previous research has showed that managers’ perceived (horizontal) vertical lines of management control to be operating according to the (innovative) traditional conceptualization of management information and control systems. Hospitals are complex organizations in which activities and functional structures have emerged in which interaction and lateral relations are key to managing optimal performance. Healthcare managers must face the challenge to go beyond traditional control systems based on hierarchical and vertical lines, and adopt more horizontal management control systems, which are focused on managing complexity and interaction between healthcare units and activities.

Based on previous literature, the objective of this paper is to analyse the direct relationship between the clinical experience and the perceived utility of information and horizontal control systems in hospitals. We also analyse the indirect effect of clinical experience in financial and operational performance through the perceived utility of information and horizontal control.

## 3. Materials and Methods

### 3.1. Data Collection

The sample was selected from a public hospitals’ database developed by the Brazilian Health Ministry (Ministério da Saúde). Under the new public management paradigm, Brazilian healthcare authorities design management control systems and encourage hospitals to use them to support quality in healthcare services at a lower cost. In this regard, small hospitals are likely to lack a minimal managerial structure and the resources to comply with the recommendations [20,21]. As such, we employed a procedure of stratified sampling by size, selecting only hospitals that reported a minimum of 150 beds and provided an email address and contact telephone number.

Following Dillman et al. [22] procedure, data were collected from June to November 2017 through a cross-sectional survey focused on top managers in 364 hospitals. First, we emailed the hospital CEOs, briefly explaining the project and asking for their collaboration. Next, we sent the survey, along with comprehensive information about the project, to the Scientific and Ethic committees at the 66 hospitals that agreed to participate. These 66 hospitals are distributed throughout the five regions of Brazil, constituting 18% of the target population (see the representativeness of the sample in Table 1 Panel A). The response rate is similar to the percentages reported in recent studies in the area [23]. Finally, each committee issued a formal ethics-based approval of this study and designated a contact person that forwarded the online survey to their top managers. We received 150 questionnaires in total; however, some lacked necessary data to test the research model of this study. Therefore, the final sample for this study resulted in 56 questionnaires. We do not find significant differences between the mean scores of the main variables for early and late respondents (Table 1 Panel B).

### 3.2. Measurement of Variables

Clinical experience of managers was measured by number of years of clinical experience in public hospitals and other types of health institutions [24].

Perceived utility of management information was measured using a five-point Likert scale (1: low utility; 5: high utility) for several items, including the utility of forecasted vs. historical information, long-term vs. short-term information, non-financial vs. financial information, and external vs. internal information [24,25].

Horizontal management control was measured using a five-point Likert scale (1: strongly disagree; 5: strongly agree). Managers were asked about the use of management control for (i) monitoring team activities done directly by peers at the same organizational level; (ii) monitoring team activities done directly by peers in the same work environment; and (iii) monitoring team activities depending on the specification, measurement, and assessment defined by someone outside the team.

Hospital performance measurement was measured using a five-point Likert scale (1: well below the average; 5: well above the average). Managers were asked to indicate their perception about the performance of their hospital compared to other hospitals of similar size and function in terms of (i) clinical costs, (ii) capacity to get resources, (iii) clinical reputation, (iv) university teaching programmes, (v) research, and (vi) quality of care. Items (i) and (ii) were used to measure financial performance. Items (iii) and (iv) were used to measure operational performance.

## 4. Results

Table 1—Panel C displays the demographic data of the sample. Validity and reliability tests for all the variables are shown in Table 2.

Cronbach’s α of constructs exceeded or met the suggested cut-off value of 0.7, and the composite reliability (CR) values exceeded the suggested cut-off value of 0.8. These results indicated that our measurement met the reliability criteria. Hair et al. [26] suggest using the mean extracted variance (AVE) as a measure of convergent validity. Table 2 shows that AVEs ranged from 0.65 to 0.93, exceeding the cut-off value of 0.6, suggesting satisfactory convergent validity [26]. In general, all constructs in this study exhibited sufficient convergent and discriminant validity.

We used the Partial Least Square (PLS) method to analyse the relationships between our research variables. Similar to equation structure procedures, PLS is a second-generation statistical technique that aims to maximize the explained variation for each relation. The path coefficients are identical to the betas produced in regression analysis. PLS is especially pertinent to this study, because it does not require normally distributed data, makes minimal data assumptions, and is appropriate for small sample sizes [27,28]. For the analysis of the structural model, the bootstrapping method (5000 samples) was used, according to Hair et al. [26].

The path coefficients between clinical experience and perceived utility of management control information is negative, with strong statistical significance (β = −0.342, *p* < 0.01). Table 3 presents the coefficients (β) of the relations.

The results reported in Table 3 indicate a positive relationship between perception of the usefulness of management control information and financial performance (β = 0.392, *p* < 0.01); however, the relationship with operational performance is not significant. These findings show that the perception of the usefulness of information acts as an intervening variable in the relationship between clinical experience and financial performance, explaining approximately 17.5% of the variance in financial performance.

There is also a positive association between horizontal management control and operational performance (β = 0.347, *p* < 0.10). The results show that control of team activities is related to operational performance but not to financial performance (β = 0.089, *p* > 0.10).

Table 4 presents the results of eight sub-group analyses performed as an additional analysis and also as a test of the stability of our results. For that purpose we use gender, age, academic education, and professional position as splitting variables. Every model respects the recommended goodness-of-fit threshold indices. The results obtained for all relationship are consistent with our primary analysis. Moreover, the additional test indicates that (1) The effect of clinical experience on perceived utility information is observed particularly for male, young, postgraduated, and administration-related managers; (2) The relationship between horizontal management control and operative performance is limited to postgraduated managers; and (3) The effect of perceived utility information on financial performance is observed particularly for female, young, postgraduated, and clinical-related managers.

## 5. Discussion 

The general objective of this paper was to increase our understanding of the mechanisms that enhance performance in hospitals. We analysed the managerial information and management control as mediating variables between the clinical experience of managers and organizational performance. As public hospitals are organizations that spend more than 10% of their budget on processing and using managerial information, understanding how managers use management control seems important, especially when management control is key to managing hospital services and implementing healthcare policies and strategies. Overall, our paper shows that managers’ clinical experience affects management control and healthcare services delivered by the hospital. Thus, managers with greater clinical experience perceive narrow-scope management information to be more useful than broad-scope information for managing hospitals [29]. Narrow-scope information, such as internal or short-time oriented information, is focused on critical aspects of daily services and functions carried out in hospitals. These results also indicate that the utility of particular information should be evaluated considering the characteristics of the directors who will use it. It may make little sense to provide a specific type of information to managers whose background is such that they will tend to ignore it. This does not mean that the information cannot be used by others, only that it may not be useful to these managers in particular.

Our findings also show that managers’ clinical experience is not related to the adoption of horizontal management control, which lets hospitals better identify and react to uncertainties in a timely way. Horizontal management control lets managers discuss managerial information on critical services and functions across all hierarchical levels in the hospital. Several studies in healthcare management argue that adoption of horizontal control requires a combination of cooperation, coordination, and autonomy, which stimulates flexible and fluent relationships across the hospital, thus enhancing organizational performance [29,30]. In this vein, our findings show that perceived utility of management information positively affects financial, rather than operational, performance in hospitals. Our results also show that horizontal control management is positively related to operational performance rather than financial performance in a hospital. Overall, these results provide evidence that the combined use of managerial information and horizontal control is needed to enhance hospital performance.

Our findings are consistent with the theoretical development that clinical managers are more accustomed to the use of internal and adaptable systems to manage and control organizations [31,32]. Managers in hospitals may use narrow-scope management information, such as short-term and internal oriented information, to allocate resources in the hospital and provide information, on time, on a need-to-perform basis, to employees across hospitals.

## 6. Conclusions

Our results lead us to conclude that the clinical experience of hospital managers may affect the use of managerial information systems and thus hospital performance. We can conclude that the focus of the perceived utility of management information on narrow-scope characteristics such as short-term, financial, internal, and historical information has an important intermediate role in the relationship between clinical experience of managers and hospital financial performance. This role has practical implications due to the likely effect of clinical experience in coaching hospital employees in all hierarchical levels and functions. As the clinical experience of managers affects managerial information perception, healthcare authorities, who appoint hospital managers, should not only encourage managers to gain clinical experience but also support training in dealing with management information in order to gain efficiency in carrying out activities, autonomy of work, and coordination in clinical service delivery. An increase in the clinical side of management experience may pay off, as it allows clinical managers to use managerial information in a way that could facilitate the way in which they address the uncertainty and complexity of healthcare services. In this way, managers can actively stimulate interaction among employees in hospitals. All of this may require that the healthcare authorities pay increased attention to the proper alignment between managers’ background and a hospital’s needs and objectives.

Like any empirical study, this research has its limitations. There are some limitations related to the questionnaire, such as the use of perceptual measures and the use of non-random sampling [24]. This may be a particular case of the “common method” problem, associated with the use of a single questionnaire with self-response measures [22]. In addition, two other limitations are causality and focus on a single sector. First, causality cannot be evaluated through the use of cross-sectional studies such as this research. Second, focusing on a single industry allows us to acquire a deep knowledge of the sector, which is very useful for reliably interpreting the statistical findings. However, the main disadvantage of relying on a single industry is that it is not very appropriate to generalize the findings, although this does not mean that the results cannot be extended to other sectors. Clearly, the empirical verification of the research objectives of this paper in other industries is essential to give greater external validity to the results. Future research could also use longitudinal data that could specify the direction of the statistical associations observed in this study. Since this research used only questionnaires to collect the data, future studies could also use qualitative methods. For example, the actual observation of managers using management information systems could provide valuable data about the use of these systems rather than just asking questions about their perceptions.

## Figures and Tables

**Table:** 

Panel A: Representativeness of the Sample (N = 66)				
Number of Beds	Sample		Population	
	Number	%	Number	%
251–250	43	65.15	378	62.90
251–350	13	19.70	129	21.46
351–450	7	10.60	45	7.49
451+	3	4.55	49	8.15

**Table:** 

Panel B: Comparison of Main Variables for Early and Late Respondents (N = 56)			
Variable	Mean of Construct Values		
	Early Respondents (First 25 Responses Received)	Late Respondents (Last 25 Responses Received)	
CE	2.68	2.30	t = 0.788 (*p* = 0.435)
PUI	2.88	3.32	t = −1.760 (*p* = 0.081)
HMC	2.60	3.16	t = −2.657 (*p* = 0.010)
FP	3.06	3.08	t = −0.082 (*p* = 0.935)
OP	3.25	3.48	t = −1.210 (*p* = 0.229)

**Table:** 

Panel C: Descriptive Statistics for Demographic Variables (N = 56)			
		N	(%)
Professional Position	Chief Executive Officer	11	19.64
	Clinical Director	7	12.50
	Nursing Director	7	12.50
	Other Chief Officers	31	55.36
Gender	Female	19	33.93
	Male	37	66.07
Academic Education	Graduate	11	19.64
	MBA	28	50.00
	Master	11	19.64
	Doctorate	6	10.72
Age (Years)	21–30	1	1.79
	31–40	11	19.64
	41–50	19	33.93
	51–60	16	28.57
	Over 61	9	16.07

CE: Clinical experience, PUI: Perceived utility of management information, HMC: Horizontal management control, FP: Financial performance, OP: Operational performance.

**Table 2:** Results the factor loading, reliability, and validity of variables.

Variables	Item	Factor Loading	Cronbach’s	CR	AVE
Clinical experience	CE1	0.937	0.820	0.917	0.846
	CE2	0.902			
Perceived utility of management information	PUI1	0.949	0.976	0.982	0.932
	PUI2	0.962			
	PUI3	0.982			
	PUI4	0.968			
Horizontal management control	HMC1	0.804	0.732	0.848	0.650
	HMC2	0.841			
	HMC3	0.773			
Financial performance	FP1	0.771	0.667	0.847	0.736
	FP2	0.937			
Operational performance	OP1	0.702	0.854	0.899	0.693
	OP2	0.896			
	OP3	0.885			
	OP4	0.832			

**Table 3:** PLS structural model: path coefficients, t-statistics.

Dependent Variables	Independent Variables		
	CE	PUI	HMC
Perceived utility of management information	0.050 (0.218)	-	-
Horizontal management control	−0.342 (2.582) ***	-	-
Financial performance	-	0.392 (3.006) ***	0.089 (0.499)
Operational performance	-	0.074 (0.345)	0.347 (1.654) *

CE: Clinical experience, PUI: Perceived utility of management information, HMC: Horizontal management control. Each cell reports the path coefficient (t-value). * *p* < 0.10; *** *p* < 0.01.

**Table 4:** Results of the sub-group analyses.

Variables	Path Coefficients							
	Gender		Age		Academic Education		Professional Position	
	Male	Female	20 to 50	50+	Graduate	Postgraduate	General/Admin ^†^	Clinical/Nursing ^†^
CE → HMC	−0.138	−0.138	−0.189	0.540	0.757	−0.019	0.134	0.102
CE → PUI	−0.483 ***	−0.330	−0.553 ***	−0.108	0.235	−0.445 ***	−0.331 *	−0.343
HMC → FP	0.103	−0.039	0.086	0.128	0.396	0.079	0.155	−0.040
HMC → OP	0.256	0.412	0.404	0.335	0.500	0.518 ***	0.416	0.416
PUI → FP	0.276	0.623 ***	0.428 **	0.318	0.474	0.359 **	0.353	0.651 *
PUI → OP	−0.202	0.399	−0.017	0.124	0.298	−0.074	0.107	0.136

^†^ Managers. CE: Clinical experience, PUI: Perceived utility of management information, HMC: Horizontal management control. Each cell reports the path coefficient. * *p* < 0.10; ** *p* < 0.05; *** *p* < 0.01.
